# Publisher Correction: A ubiquitin chain-feeding mechanism for BRCA1-A.

**DOI:** 10.1038/s41467-026-77841-1

**Published:** 2026-09-21

**Authors:** Andrea G. Murachelli, Farid El Oualid, Titia K. Sixma

**Affiliations:** 1 Netherlands Cancer Institute and Oncode Institute, Amsterdam, the Netherlands; 2 UbiQ Bio B.V., Amsterdam, the Netherlands

**Keywords:** Cryoelectron microscopy, Enzyme mechanisms, Chemical tools

Correction to: *Nature Communications* https://doi.org/10.1038/s41467-026-75797-w, published online 11 August 2026

In the version of this article initially published, the Supplementary Information file was incomplete and is now amended in the HTML version of the article.

