# Supplementary material for: A ubiquitin chain-feeding mechanism for BRCA1-A
Source: Nat Commun. 2026 Aug 11;17:8092. doi: 10.1038/s41467-026-75797-w (PMC13462734; doi:10.1038/s41467-026-75797-w)
Supplement: Supplementary file 2 [file 41467_2026_75797_MOESM2_ESM.pdf]

Description of Additional Supplementary Files

File name: Supplementary Movie 1

Description: **BRCA1-A closes upon substrate engagement.** Morph between the apo and polyUb$_{ATA}$-bound BRCA1-A structures. PolyUb$_{ATA}$ omitted for clarity.

File Name: Supplementary Movie 2

Description: **Active sites engage simultaneously, but cleave in alternation.** The active site in State P cannot transition to State C until cleavage has occurred at the opposing State C site.

File name: Supplementary Movie 3

Description: **BRCA1-A alternates between open and closed forms.** Morph between the apo, open polyUbATA-bound, and closed polyUbATA-bound structures, illustrating the conformational range of BRCA1-A. PolyUbATA omitted for clarity

File Name: Supplementary Movie 4

Description: **Arm closure shifts Ub$_{elbow}$ toward the active site.** Morph between the open and closed polyUbATA-bound structures, illustrating the forward shift of Ub$_{elbow}$ upon arm closure.

File Name: Supplementary Movie 5

Description: **Arm dynamics coordinate substrate feeding and cleavage.** Animated representation of the mechanistic model presented in Figure 6.
