# Supplementary material for: A ubiquitin chain-feeding mechanism for BRCA1-A
Source: Nat Commun. 2026 Aug 11;17:8092. doi: 10.1038/s41467-026-75797-w (PMC13462734; doi:10.1038/s41467-026-75797-w)
Supplement: Supplementary file 4 [file 41467_2026_75797_MOESM9_ESM.pdf]

nature portfolio

Peer Review File

A ubiquitin chain-feeding mechanism for BRCA1-A

Corresponding Author: Professor Titia Sixma

This file contains all reviewer reports in order by version, followed by all author rebuttals in order by version.

Version 0:

Reviewer comments:

Reviewer #1

(Remarks to the Author)
This study presents a comprehensive structural analysis of Ly63-linked polyubiquitin chain recognition and processing by the human BRCA1-A complex, a key metallo-DUB assembly that regulates DNA double-strand break (DSB) repair. The crystal structure of the mouse BRCA1-A complex was reported earlier showing a dimer of heteropentamers composed of BRCC36 (catalytic subunit), ABRAXAS, BRE, Merit40 (BABAM1) and Rap80. These ten subunits arrange into a two-armed, arch-shaped architecture, with the two BRCC36 catalytic cores positioned at the base. Although BRCA1-A was known to prefer long Lys63-linked polyubiquitin chains, the structural basis for this selectivity, and how substrate recognition by the arms of the complex is coordinated with catalysis at the base, had remained unsolved.
Using non-cleavable analogues of polyubiquitin chains, the authors have managed to capture cryo-EM snapshots of different dynamic states of the BRCA1-A complex engaged with its substrates of different lengths. Several distinct states of the complex were captured which allowed the authors to postulate a novel chain-feeding model of the enzyme that proposes an asymmetric recognition and alternating cleavage at the two active sites. In this model, anchoring interactions in the arms guide and stabilize distal ubiquitins, while the chain is sequentially fed toward the catalytic BRCC36 subunits for processive trimming of Lys63-linked chains into shorter fragments.
Biochemical assays looking at chain cleavage and biophysical analysis of substrate binding were used to support the structural studies and some aspects of the model.
Some of the strengths of their manuscript are:
• The authors introduce a zinc-chelating thiol warhead (4-amnio-3-thiobutyric acid, ATA) to create novel activity-based ubiquitin probes (diUbATA, oligoUbATA and polyUbATA) for JAMM-family DUBs which they used to capture different conformational and catalytic states of the BRCA1-A complex. The synthetic strategy can be adapted as a general strategy for the generation of similar probes of any other linkage type of polyubiquitin chains. In this part, they first synthesized diUbATA via solid phase peptide synthesis and later use this dimer as a building block for enzymatically extension into oligo and polyubiquitin probes containing alternating non-cleavable ATA and native isopeptide linkers.

• The authors present the first high-resolution cryo-EM structures of BRCA1-A in multiple substrate-bound states. Interestingly, they discovered that the otherwise symmetric BRCC36 dimer binds diUbATA asymmetrically. The diUb substrate analog was captured in a non-productive state (P-state, which they refer to as the precatalytic state) in the active site of one BRCC36 monomer and in a catalytically competent state (C-state) in the other, a unique and mechanistically insightful finding.

• The authors also show how the BRCA1-A complex alternates between open and closed conformations to coordinate chain feeding and processivity, thereby establishing BRCA1-A as a chain-trimming enzyme with preference for K63-poly Ub chains.

• The catalytically competent state of the BRCA1-A complex clearly shows how the ABRAXAS-finger (loop residues 39-57) from one protomer extends into the active site of the opposite pentamer, and the mechanistic importance of this in facilitating catalysis.

• The authors propose an original insightful hypothesis that couples the rope-and-pulley proposed mechanism with conformational dynamics observed upon substrate engagement.

Weaknesses

• One concern is the lack of cell-based experiments to validate the findings. The authors have not tested how their key structural findings from the different substrate-bound states they discuss in the manuscript impact BRCA1-A recruitment or DSB repair. Although the structures and biochemical data are compelling, the proposed mechanism remains conjectural in the absence of validation experiments in cell. (see below, point 1)

• Lack of rigorous kinetic analysis to validate their claims of processivity is another concern. (see below, point 2)

This is a strong and compelling manuscript, well written, clearly organized, and nicely illustrated. The structural findings are novel, and the mechanistic interpretations are thoughtful and generally well supported by the cryo-EM data. However, several issues limit the impact of the current report. In particular, the lack of cell-based validation assays and the absence of more rigorous kinetic analyses with chains of defined length weaken the functional conclusions. I would urge the authors to consider the points below and provide additional biochemical and cellular data towards the improvement of the manuscript.

1. The authors may consider validating some of their key structural findings in a cell-based assay. For example, the ABRAXAS finger making in trans contribution to catalysis is interesting. A functional assay examining the effect of the triple-alanine fingertip mutant on BRCA1-A recruitment or DSB repair would directly address whether this mechanism operates in a cellular context.

2. I think the chain processing assays should be performed with a single type of ployUb chain (of a defined length) instead of the mixture. For example, the data shown in Figure 4G is hard to interpret along the lines suggested by the authors. The authors should consider performing this sort of chain-processing assays using defined substrates (e.g., pure K63-Ub6 vs. shorter chains).

3. Arg89 seems important in the distinction between the P and C states. An activity assay testing an R89 mutant would help clarify if this residue contributes directly to catalysis.

4. The activity assay gels should have loading controls (western blot for one of the BRCA1-A components, for example).

5. The authors need to justify why they think the P state is more likely to be a pre-catalytic state. Also, the thiol warhead seems to coincide between the C and P state (Figure S3A). Why, then, is the P state considered catalytically incompetent?

6. Indicating the positions of the Lys63 and C-terminal ends of Ubelbow and Ubwrist in the model would help orient the polyubiquitin chain.

7. Please provide some more clarity on Figure 1E. The legend says it is coomassie stained but the figure indicates anti-Ub as if it were a western blot.

8. Please check E and D’s again. (In figure 2C, it should be E33 and somewhere else in the text the 88th residue is referred to as E88.)

9. Please include the native isopeptide linkage (Lys63-Gly76) in Figure 1B, so one can appreciate how the probe mimics the native substrate.

10. Please label UEV domains in BRE, as is done for other components, in Figure 1A. It may also help to explicitly relate the elbow and wrist binding surfaces to specific regions of the UEV domains in the text.

Reviewer #2

(Remarks to the Author)

This manuscript by Murachelli et al. presents a comprehensive mechanistic study of the BRCA1-A complex, a multi-subunit metallo-deubiquitinase with specificity for K63-linked polyubiquitin chains. The development of a diubiquitin-based activity probe (diUbATA) featuring a zinc-chelating thiol warhead is a significant methodological advance, providing a versatile tool for interrogating metallo-DUBs that, unlike cysteine-based DUBs, have been challenging to target. The subsequent enzymatic polymerization of this probe into chains with alternating cleavable and uncleavable linkages is particularly clever, enabling the capture of various catalytic intermediates by cryo-EM. The structures reveal several intermediate states of the enzyme and highlight the role of allosteric regulation by the ABRAXAS subunit, as well as avidity-driven recognition of longer ubiquitin chains through auxiliary binding sites on the BRE subunit. The proposed “chain-feeding” model provides a plausible explanation for the complex’s preference for longer ubiquitin chains and its processive cleavage behavior. The work is methodologically innovative and offers significant insights into the mechanism of a biologically important deubiquitinating enzyme. Below are several points that could be addressed to further strengthen the study.

The design and application of the diUbATA probe featuring a zinc-chelating thiol warhead represent a notable methodological innovation. To maximize its impact and utility for the scientific community, it would be highly beneficial to include a more detailed synthetic protocol in the Supplementary Information. This could encompass intermediate yields,

purification outcomes, and critical quality control checkpoints (e.g., HPLC and MS characterization data), which would significantly enhance the reproducibility of this valuable tool. The current work marks a critical step forward in the development of activity-based probes for zinc-dependent deubiquitinases. Given its significance in chemical biology, additional details regarding experimental reproducibility, thorough characterization, and potential adaptations would merit further elaboration in the manuscript.

Furthermore, the authors may consider expanding the discussion to highlight the broader potential of this probe strategy. For instance, it could be applied to investigate the hydrolysis mechanisms of other ubiquitin linkages by different JAMM-family deubiquitinases, or even be utilized in complex environments such as cell lysates for activity-based profiling. When adapting this strategy to different ubiquitin chain types, key chemical and structural considerations—such as the geometry of the active site and the spatial orientation of the catalytic zinc ion—will be essential.

The polyUbATA chains feature a unique topology, with alternating cleavable and non-cleavable linkages. Whether this design might influence the conformational states captured in their structures? Specifically, would the presence of uncleavable linkages predispose the complex to populate or stabilize certain conformational states (e.g., State C*, or the closed form) that differ from its behavior on native, fully cleavable chains? Furthermore, the structural basis for how the BRCC36 active site and the surrounding exosites distinguish between a cleavable isopeptide bond and the non-cleavable ATA warhead remains an intriguing question. Some commentary on the observed interactions that might facilitate this discrimination would strengthen the mechanistic interpretation.

The conclusions drawn from the BRE ubiquitin-binding site mutants are compelling, yet the possibility that these mutations alter the overall structural integrity or stability of the complex—rather than exclusively impacting binding affinity—has not been explicitly addressed. Supplementary controls, such as size-exclusion chromatography or thermal shift assays, would help rule out gross structural perturbations.

The proposed "chain-feeding" model is compelling and supported by an ensemble of conformational states. That said, the model is based largely on static snapshots. The authors may wish to discuss how future work (such as single-molecule, virtual simulation) could provide more direct evidence for substrate translocation and cleavage dynamics. In addition, systematic analysis of cleavage rates across ubiquitin chains of defined lengths (e.g., Ub3–Ub8) would help clarify the minimal chain length required for avidity and processivity.

While the in vitro findings are robust, their connection to cellular function could be further strengthened. The authors propose that BRCA1-A acts as a chain-trimming enzyme in DNA repair, but the discussion could be extended to include how the observed mechanisms may influence pathway choice (e.g., homologous recombination vs. non-homologous end joining) in a physiological condition.

Given the high density of specialized abbreviations (e.g., diUbATA, UbD, UbP, State C/P) and complex structural descriptions, the text is inherently challenging to parse. While the definitions are provided upon first use, the readability might be further enhanced by consolidating these terms in a supplementary table or glossary. A careful review to ensure that pronouns and modifiers have unambiguous antecedents in long sentences could also improve clarity.

The synthesis of the diUbATA probe and its enzymatic polymerization into chains is central to this study. To properly credit the original work and aid in reproducibility, it would be helpful to include a citation to the primary reference describing the native chemical ligation strategy used in its preparation.

In conclusion, this study represents a substantial contribution to the fields of ubiquitin signaling and DNA repair. The novel probe development, the high-resolution structural insights into BRCA1-A's mechanism, and the elegant model for processive chain cleavage are highly compelling. Addressing the points raised above would further solidify the findings and maximize the impact of this impressive work.

Version 1:

Reviewer comments:

Reviewer #1

(Remarks to the Author)
This manuscript is much improved in its current form, particularly with the addition of new results, such as the use of better defined polyubiquitin chain substrates in the deubiquitination assays and the quantitative analysis of substrate depletion and accumulation of products of different lengths. The authors made a sincere effort to perform the cellular experiments suggested by this reviewer. Although these experiments were ultimately unsuccessful, their absence does not seriously affect the overall quality, rigor or the conclusions of the study. All my other concerns and comments have been adequately addressed.

Reiterating my comments from the previous round of review, I believe this is a well written and technically solid manuscript that provides several novel insights into the deubiquitinase activity of the BRCA1-A complex and its mechanism of action. I believe this study will be of considerable interest to both the ubiquitin and DNA damage response communities and is likely to have a meaningful impact in the field. I therefore have no hesitation in recommending its publication in this journal.

(Remarks on code availability)
NA

Reviewer #2

(Remarks to the Author)

The authors have appropriately addressed my previous concerns. The revised manuscript is scientifically sound and I support its publication

(Remarks on code availability)

Open Access This Peer Review File is licensed under a Creative Commons Attribution 4.0 International License, which permits use, sharing, adaptation, distribution and reproduction in any medium or format, as long as you give appropriate credit to the original author(s) and the source, provide a link to the Creative Commons license, and indicate if changes were made.
In cases where reviewers are anonymous, credit should be given to 'Anonymous Referee' and the source.
The images or other third party material in this Peer Review File are included in the article's Creative Commons license, unless indicated otherwise in a credit line to the material. If material is not included in the article's Creative Commons license and your intended use is not permitted by statutory regulation or exceeds the permitted use, you will need to obtain permission directly from the copyright holder.
To view a copy of this license, visit https://creativecommons.org/licenses/by/4.0/

REVIEWER COMMENTS

We thank the reviewers for their thorough and constructive comments.
Based on these comments we have:

- Expanded the description of the generation of the ATA probe (Figure S1B, C)
- Added quantification of activity assays by densitometry, and plotted the change in Ub species over time to allow easier reading of the activity assays (Fig. 1B, 2E, 2F, 3D, 3E, 4G; Supplementary Fig. 1A, 1B, 4H, 4I, 5F). Loading controls are present on all gels.
- Used these quantifications to derive cleavage kinetic data on pools of ubiquitin chains with defined short vs long chain-lengths (new Fig 1B; Supplementary Fig. 1A, 1B).
- Added new mutant activity data on R89A (added to Fig 2E, 2F)
- Clarified BRE binding sites relative to ancestral UEVs (updated Fig 1A)
- Improved description of state P, with additional panel (Fig 3B)
- Added a stability analysis for mutants (gel filtration and melting point analysis) (New Supplementary Figure 6)
- Added a reflection on the possible consequences of the chain trimming ability of BRCA1-A (reviewer 2). Direct testing of cellular consequence (reviewer 1) was unfortunately not within the scope of the manuscript as we found that the phenotype of Abraxas knockouts reverted too rapidly to be studied.

Reviewer #1 (Remarks to the Author):

This study presents a comprehensive structural analysis of Ly63-linked polyubiquitin chain recognition and processing by the human BRCA1-A complex, a key metallo-DUB assembly that regulates DNA double-strand break (DSB) repair. The crystal structure of the mouse BRCA1-A complex was reported earlier showing a dimer of heteropentamers composed of BRCC36 (catalytic subunit), ABRAXAS, BRE, Merit40 (BABAM1) and Rap80. These ten subunits arrange into a two-armed, arch-shaped architecture, with the two BRCC36 catalytic cores positioned at the base. Although BRCA1-A was known to prefer long Lys63-linked polyubiquitin chains, the structural basis for this selectivity, and how substrate recognition by the arms of the complex is coordinated with catalysis at the base, had remained unsolved.
Using non-cleavable analogues of polyubiquitin chains, the authors have managed to capture cryo-EM snapshots of different dynamic states of the BRCA1-A complex engaged with its substrates of different lengths. Several distinct states of the complex were captured which allowed the authors to postulate a novel chain-feeding model of the enzyme that proposes an asymmetric recognition and alternating cleavage at the two active sites. In this model, anchoring interactions in the arms guide and stabilize distal ubiquitins, while the chain is sequentially fed toward the catalytic BRCC36 subunits for processive trimming of Lys63-linked chains into shorter fragments. Biochemical assays looking at chain cleavage and biophysical analysis of substrate binding were used to support the structural studies and some aspects of the model. Some of the strengths of their manuscript are:

• The authors introduce a zinc-chelating thiol warhead (4-amnio-3-thiobutyric acid,

ATA) to create novel activity-based ubiquitin probes (diUbATA, oligoUbATA and polyUbATA) for JAMM-family DUBs which they used to capture different conformational and catalytic states of the BRCA1-A complex. The synthetic strategy can be adapted as a general strategy for the generation of similar probes of any other linkage type of polyubiquitin chains. In this part, they first synthesized diUbATA via solid phase peptide synthesis and later use this dimer as a building block for enzymatically extension into oligo and polyubiquitin probes containing alternating non-cleavable ATA and native isopeptide linkers.

• The authors present the first high-resolution cryo-EM structures of BRCA1-A in multiple substrate-bound states. Interestingly, they discovered that the otherwise symmetric BRCC36 dimer binds diUbATA asymmetrically. The diUb substrate analog was captured in a non-productive state (P-state, which they refer to as the precatalytic state) in the active site of one BRCC36 monomer and in a catalytically competent state (C-state) in the other, a unique and mechanistically insightful finding.

• The authors also show how the BRCA1-A complex alternates between open and closed conformations to coordinate chain feeding and processivity, thereby establishing BRCA1-A as a chain-trimming enzyme with preference for K63-poly Ub chains.

• The catalytically competent state of the BRCA1-A complex clearly shows how the ABRAXAS-finger (loop residues 39-57) from one protomer extends into the active site of the opposite pentamer, and the mechanistic importance of this in facilitating catalysis.

• The authors propose an original insightful hypothesis that couples the rope-and-pulley proposed mechanism with conformational dynamics observed upon substrate engagement.

We thank the reviewer for their positive words.

Weaknesses

• One concern is the lack of cell-based experiments to validate the findings. The authors have not tested how their key structural findings from the different substrate-bound states they discuss in the manuscript impact BRCA1-A recruitment or DSB repair. Although the structures and biochemical data are compelling, the proposed mechanism remains conjectural in the absence of validation experiments in cell. (see below, point 1)

• Lack of rigorous kinetic analysis to validate their claims of processivity is another concern. (see below, point 2)

This is a strong and compelling manuscript, well written, clearly organized, and nicely illustrated. The structural findings are novel, and the mechanistic interpretations are thoughtful and generally well supported by the cryo-EM data. However, several issues limit the impact of the current report. In particular, the lack of cell-based validation

assays and the absence of more rigorous kinetic analyses with chains of defined length weaken the functional conclusions. I would urge the authors to consider the points below and provide additional biochemical and cellular data towards the improvement of the manuscript.

1. The authors may consider validating some of their key structural findings in a cell-based assay. For example, the ABRAXAS finger making in trans contribution to catalysis is interesting. A functional assay examining the effect of the triple-alanine fingertip mutant on BRCA1-A recruitment or DSB repair would directly address whether this mechanism operates in a cellular context.

> Indeed, we did try this. We generated RPE1 ABRAXAS knockout cells and, in collaboration with the lab of Sylvie Noordermeer, we planned rescue experiments with wild-type or Abraxas3A finger mutants. Multiple independent ABRAXAS−/− clones initially exhibited a mild DNA damage sensitivity phenotype. However, after several passages, the cells appeared to compensate and reverted to a wild-type–like phenotype, suggesting the presence of adaptation in a mechanism that remains unclear. We consider it important to resolve this phenomenon, and the Noordermeer lab will pursue this further. However, given multiple unsuccessful attempts in that lab—both within this collaboration and in earlier work—we anticipate that this will require substantial additional effort and is therefore beyond the scope of the present study.

2. I think the chain processing assays should be performed with a single type of ployUb chain (of a defined length) instead of the mixture. For example, the data shown in Figure 4G is hard to interpret along the lines suggested by the authors. The authors should consider performing this sort of chain-processing assays using defined substrates (e.g., pure K63-Ub6 vs. shorter chains).

> Unfortunately, we believe precise kinetic analysis as suggested is not yet technically possible. There is currently no established commercial supplier for chains of defined length greater than Ub_4, and to our knowledge the longest chains of defined length presented in the literature are Ub_5 (Abdul Rehman *et al.,* Mol.Cell 2025). Moreover, a detailed model accounting for the full complexity of the cleavage mechanism (length-dependent chain affinity, avidity, processivity, alternate cleavage, plus endo- and exo-cleavage with variable length products) would require a number of parameters far exceeding the available observables. Under these conditions, any fitted model would be underdetermined and therefore unable to provide meaningful insight.
> Therefore, to address this point, we have used size exclusion, to create pools of chains with different length: we separated chains larger Ub_{10} or greater (long chains) from chains of length Ub_6 and shorter (short chains). These were used to derive apparent decay rates for long chains (Ub_{15+}), intermediate chains (Ub_{4-6}) and short chains (Ub_2, Ub_3) respectively (new Fig. 1B; Supplementary Figure 1A, 1B) and we could show that these are significantly different. In addition, we repeated all digestion experiments, including Fig. 4G, with long or short chains

and/or we quantified all Ub species separately (Fig 1B/S1A, S1B; 2D; 3D; 4G/S4H; S5F).

3. Arg89 seems important in the distinction between the P and C states. An activity assay testing an R89 mutant would help clarify if this residue contributes directly to catalysis.

We have added new experiments to address this (revised Fig 2D,2E): Mutation of BRCC36 Arg89Ala results in a strong catalytic impairment (estimated at ~256–512-fold). This effect is more pronounced than that observed for the ABRAXAS finger mutant (ABRAXAS^{3A}), indicating that the role of Arg89 extends beyond its interaction with the ABRAXAS finger and includes a direct contribution to catalysis. We have revised Fig. 2D to display the effects of the ABRAXAS^{3A} and BRCC36^{R89A} mutations side by side and updated the text accordingly to describe the BRCC36 mutant.

4. The activity assay gels should have loading controls (western blot for one of the BRCA1-A components, for example).

We had inadvertently omitted the loading controls for Fig. 2D in the submitted manuscript. All time-course figures now include a control lane corresponding to either 1 or 5 µL of the 1 µM stock used in the experiment.

5. The authors need to justify why they think the P state is more likely to be a pre-catalytic state. Also, the thiol warhead seems to coincide between the C and P state (Figure S3A). Why, then, is the P state considered catalytically incompetent?

In State P, displacement of UbP opens the backside of the reaction chamber to bulk solvent, likely perturbing the positioning and chemical potential of the catalytic water (represented here by the thiol). On the opposite side, the scissile bond is also exposed to solvent due to displacement of the lid residue Arg89. Although we cannot formally exclude that cleavage might occur in State P, the data support catalytic incompetence.
We have changed the text to explicitly mention the above, and added figure 3B to show the opening of the reaction chamber in state P.

6. Indicating the positions of the Lys63 and C-terminal ends of Ubelbow and Ubwrist in the model would help orient the polyubiquitin chain.

Thanks, we have done this in Figure 1G.

7. Please provide some more clarity on Figure 1E. The legend says it is Coomassie stained but the figure indicates anti-Ub as if it were a western blot.

Yes, thank you it is indeed a western blot, there was an error in the legend. This has now been resolved.

8. Please check E and D's again. (In figure 2C, it should be E33 and somewhere else in the text the 88th residue is referred to as E88.)

> Thanks, we have corrected this error.

9. Please include the native isopeptide linkage (Lys63-Gly76) in Figure 1B, so one can appreciate how the probe mimics the native substrate.

> We have added a representation of the native isopeptide bonds above the ATA linkage in Fig. 1C.

10. Please label UEV domains in BRE, as is done for other components, in Figure 1A. It may also help to explicitly relate the elbow and wrist binding surfaces to specific regions of the UEV domains in the text.

> Rabl *et al.* describe BRE as the fusion of three Ubiquitin Conjugating Enzyme superfamily domains (CATH Superfamily 3.10.110.10). Current databases instead indicate the fold as "BRE-like" (or BABAM2-like). To reconcile the two approaches, we have added approximate boundaries of the ancestral domains within the BRE domain cartoon in figure 1A, and updated the legend to reflect that.
> Neither wrist nor elbow ubiquitin bind on surfaces commonly used by E2s to recognise ubiquitin. We added this information to the main text in the section where the binding sites are first described.

Reviewer #2 (Remarks to the Author):

This manuscript by Murachelli et al. presents a comprehensive mechanistic study of the BRCA1-A complex, a multi-subunit metallo-deubiquitinase with specificity for K63-linked polyubiquitin chains. The development of a diubiquitin-based activity probe (diUbATA) featuring a zinc-chelating thiol warhead is a significant methodological advance, providing a versatile tool for interrogating metallo-DUBs that, unlike cysteine-based DUBs, have been challenging to target. The subsequent enzymatic polymerization of this probe into chains with alternating cleavable and uncleavable linkages is particularly clever, enabling the capture of various catalytic intermediates by cryo-EM. The structures reveal several intermediate states of the enzyme and highlight the role of allosteric regulation by the ABRAXAS subunit, as well as avidity-driven recognition of longer ubiquitin chains through auxiliary binding sites on the BRE subunit. The proposed "chain-feeding" model provides a plausible explanation for the complex's preference for longer ubiquitin chains and its processive cleavage behavior. The work is methodologically innovative and offers significant insights into the mechanism of a biologically important deubiquitinating enzyme. Below are several points that could be addressed to further strengthen the study.

ω The design and application of the diUbATA probe featuring a zinc-chelating thiol

warhead represent a notable methodological innovation. To maximize its impact and utility for the scientific community, it would be highly beneficial to include a more detailed synthetic protocol in the Supplementary Information. This could encompass intermediate yields, purification outcomes, and critical quality control checkpoints (e.g., HPLC and MS characterization data), which would significantly enhance the reproducibility of this valuable tool. The current work marks a critical step forward in the development of activity-based probes for zinc-dependent deubiquitinases. Given its significance in chemical biology, additional details regarding experimental reproducibility, thorough characterization, and potential adaptations would merit further elaboration in the manuscript.

> We thank the reviewer for these positive comments. We have added a more extended method section on the development of the probe, including intermediate synthesis steps with MS characterization (Figure S1D, E).

ω Furthermore, the authors may consider expanding the discussion to highlight the broader potential of this probe strategy. For instance, it could be applied to investigate the hydrolysis mechanisms of other ubiquitin linkages by different JAMM-family deubiquitinases, or even be utilized in complex environments such as cell lysates for activity-based profiling. When adapting this strategy to different ubiquitin chain types, key chemical and structural considerations—such as the geometry of the active site and the spatial orientation of the catalytic zinc ion—will be essential.

> We have expanded the discussion section to explain potential use of the probe.

ω The polyUbATA chains feature a unique topology, with alternating cleavable and non-cleavable linkages. Whether this design might influence the conformational states captured in their structures? Specifically, would the presence of uncleavable linkages predispose the complex to populate or stabilize certain conformational states (e.g., State C*, or the closed form) that differ from its behavior on native, fully cleavable chains?

> This is an interesting question that cannot really be answered at this point. We do think it is likely that the ability to cleave once and then trap the complex results in state C*, as we have only observed this state with the longer chains, However, this would not mean that state does not occur with cleavable chains, just that it is harder to trap sufficient material to obtain a structure.

Furthermore, the structural basis for how the BRCC36 active site and the surrounding exosites distinguish between a cleavable isopeptide bond and the non-cleavable ATA warhead remains an intriguing question. Some commentary on the observed interactions that might facilitate this discrimination would strengthen the mechanistic interpretation.

> As mentioned in response to reviewer 1, we added a direct comparison between the ATA probe and the native linkage in Figure 1B, showing how similar these are. We also included a new figure (Fig. S2B) comparing the BRCC36 active site in

State C bound to ATA with that of AMSH-LP bound to K63-diUb; the figure legend provides a detailed description.

Briefly, the BRCC36 is correctly aligned, and the C-terminus of UbD up to the scissile bond follows essentially the same trajectory in both structures. The substantial rotation of UbP between AMSH-LP and BRCC36 precludes direct comparison of the region corresponding to Lys63. Nonetheless, the positioning and interactions of the displaced ATA carbonyl appear consistent with the conformation in the AMSH-LP structure. A more precise comparison is complicated by the fact that the reference AMSH-LP:diUb structure was determined using a point mutant lacking the catalytic zinc and water, and appears also partially distorted.

The elbow and wrist binding sites do not contact the C-terminus or Lys63 of the bound ubiquitin, suggesting that the ATA substitution does not affect binding.

We have also expanded the Discussion to include:
DiUb$_{ATA}$ appears to effectively mimic the native isopeptide linkage, allowing characterization of enzymes in a near-native state without resorting to inactive mutants, which often display disordered active sites.

ω The conclusions drawn from the BRE ubiquitin-binding site mutants are compelling, yet the possibility that these mutations alter the overall structural integrity or stability of the complex—rather than exclusively impacting binding affinity—has not been explicitly addressed. Supplementary controls, such as size-exclusion chromatography or thermal shift assays, would help rule out gross structural perturbations.

To address whether mutations would affect stability of the complex we performed nanoDSF to monitor stability of all mutants during thermal denaturation. Wild-type BRCA1-A has a melting temperature of 66°C, while all mutants melt between 62 and 67 °C. Moreover, they all eluted as single peaks of appropriate size in size-exclusion chromatography. Together, these data show that all BRCA1-A variants are well folded and stable (new Supplementary Figure 6).

ω The proposed "chain-feeding" model is compelling and supported by an ensemble of conformational states. That said, the model is based largely on static snapshots. The authors may wish to discuss how future work (such as single-molecule, virtual simulation) could provide more direct evidence for substrate translocation and cleavage dynamics.

We agree. We included the following sentence in the discussion: *Future studies, including single molecule FRET analysis, and/or trapping of additional intermediates are needed to resolve the details of this mechanism.*

In addition, systematic analysis of cleavage rates across ubiquitin chains of defined lengths (e.g., Ub3–Ub8) would help clarify the minimal chain length required for avidity and processivity.

Indeed, but as elaborated to reviewer 1, this is still technically challenging. However, we have added experiments on pooled long versus short chains to address this issue (Figure 1B, Supplementary Figure 1A, 1B). These suggest that cleavage of long chains proceeds significantly faster above the Ub_{5-6} length cutoff; This is in good agreement with the structural data, which indicate that the minimal chain length required to span the arms and engage avidity and/or processivity is ~$Ub_{6–7}$ (prior to cleavage). We added these considerations explicitly in the Results and Discussion sections as follows:

BRCA1-A preferentially acts on long polyUb chains

"BRCA1-A cleaves ubiquitin chains at markedly different rates depending on chain length. Long chains (Ub_{7+}) are processed rapidly, leading to early accumulation of shorter species ($Ub_{2–6}$). Intermediate chains ($Ub_{4–6}$) are then cleaved, followed by Ub_3 and finally Ub_2. A similar length-dependent processing order is observed when shorter chains (Ub_{2-5}) are used as substrates (Supplementary Fig. 1A). Half-lives ($t_1/_2$) estimated from the decay phase in Fig. 1B (Supplementary figure 1B) support a tiered substrate preference, with long chains (Ub_{15+}, $t_1/_2$ 0.25–0.28 min, 95% confidence interval) processed more rapidly than intermediate-length chains ($Ub_{4–6}$, $t_1/_2$ = 3.5–5.1 min) and short chains (Ub_3, $t_1/_2$ = 7–15 min; Ub_2, $t_1/_2$ = 21 – 93 min)."

BRCA1-A binds long Ub chains through avidity

"Avidity-driven substrate selection appears to explain the three-tiered cleavage kinetics observed in Fig. 1B. Chains longer than $Ub_{6–7}$ fully engage avidity and are preferentially bound and processed, leading to early accumulation of $Ub_{5–6}$. Intermediate chains ($Ub_{4–6}$), which engage avidity only partially via the elbow site, are then processed. Short chains ($Ub_{2–3}$), which interact only with the active site, are cleaved last."

Discussion
[If the cleaved chain remains bound to the arm binding sites after cleavage, BRCA1-A can close its arms, and feed the chain forward for subsequent cleavage, enabling processivity.] *This mechanism applies only to chains that fully span the arm binding sites prior to cleavage (i.e. Ub_{6+}, Fig. 4D and 5G). Accordingly, processivity, together with avidity, is expected to accelerate cleavage of long chains (Ub_{7+}) whereas intermediate ($Ub_{4–6}$) and short ($Ub_{2–3}$) chains are processed more slowly and largely in a distributive manner (Fig. 2B). Functionally, this positions BRCA1-A as a chain-shortening DUB, acting on shorter chains only after longer ones have been trimmed.*

ω While the in vitro findings are robust, their connection to cellular function could be further strengthened. The authors propose that BRCA1-A acts as a chain-trimming enzyme in DNA repair, but the discussion could be extended to include how the observed mechanisms may influence pathway choice (e.g., homologous recombination vs. non-homologous end joining) in a physiological condition.

This is an interesting question, but unfortunately the biological answer is not at all clear: one option would be that long chains recruit different factors from short chains, affecting repair pathway choice. This type of choice is seen for PCNA with one vs. more ubiquitins. It is also possible that long chains contribute to condensate formation at DNA foci. Finally, it is possible that longer chains contribute to, e.g. p97/VCP extraction/proteasome degradation and affect target presence.

We added a section to the Discussion:
"In DNA damage tolerance, choice of the pathway used to bypass DNA is influenced by the length and type of Ub chains conjugated to PCNA (reviewed in[54]), with multiple E3s and DUBs dynamically determining the balance[55–60]. How K63-polyUb chain length influences pathway choice in DSB repair is currently unknown. DUB-inactive mutants of BRCC36 lead to accumulation of K63-Ub chains[27], increased DNA resection and hyperactivation of HR repair[32], outlining a possible connection for future investigation."

ω Given the high density of specialized abbreviations (e.g., diUbATA, UbD, UbP, State C/P) and complex structural descriptions, the text is inherently challenging to parse. While the definitions are provided upon first use, the readability might be further enhanced by consolidating these terms in a supplementary table or glossary.

Such a glossary in a paper is unusual, but we have now made sure that all these terms are explained in Figure 1B, and for state C/P in Figure 2A and 2B.

A careful review to ensure that pronouns and modifiers have unambiguous antecedents in long sentences could also improve clarity.

We have tried to clarify long sentences.

ω The synthesis of the diUbATA probe and its enzymatic polymerization into chains is central to this study. To properly credit the original work and aid in reproducibility, it would be helpful to include a citation to the primary reference describing the native chemical ligation strategy used in its preparation.

We've now made explicit references [44,61] in the Methods section.

In conclusion, this study represents a substantial contribution to the fields of ubiquitin signaling and DNA repair. The novel probe development, the high-resolution structural insights into BRCA1-A's mechanism, and the elegant model for processive chain cleavage are highly compelling. Addressing the points raised above would further solidify the findings and maximize the impact of this impressive work.
